# Cryptomelane formation from nanocrystalline vernadite precursor: a high energy X-ray scattering and transmission electron microscopy perspective on reaction mechanisms

**DOI:** 10.1186/s12932-015-0028-y

**Published:** 2015-09-02

**Authors:** Sylvain Grangeon, Alejandro Fernandez-Martinez, Fabienne Warmont, Alexandre Gloter, Nicolas Marty, Agnieszka Poulain, Bruno Lanson

**Affiliations:** BRGM, 3 Avenue Guillemin, 45060 Orléans Cedex 2, France; Univ. Grenoble Alpes, ISTerre, 38041 Grenoble, France; CNRS, ISTerre, 38041 Grenoble, France; ICMN-CNRS-Université D’Orléans, 1b rue de la Férollerie, 45071 Orléans Cedex 2, France; Laboratoire de Physique des Solides, Université Paris-Sud, CNRS, UMR 8502, 91405 Orsay Cedex, France; ESRF-The European Synchrotron, 71 avenue des Martyrs, Grenoble, France

**Keywords:** Vernadite, δ-MnO_2_, Cryptomelane, Phyllomanganate, Tectomanganate, Pair Distribution Function, Bragg rod, High-energy X-ray scattering, X-ray diffraction, Transmission electron microscopy

## Abstract

**Background:**

Vernadite is a nanocrystalline and turbostratic phyllomanganate which is ubiquitous in the environment. Its layers are built of (MnO_6_)^8−^ octahedra connected through their edges and frequently contain vacancies and  (or) isomorphic substitutions. Both create a layer charge deficit that can exceed 1 valence unit per layer octahedron and thus induces a strong chemical reactivity. In addition, vernadite has a high affinity for many trace elements (e.g., Co, Ni, and Zn) and possesses a redox potential that allows for the oxidation of redox-sensitive elements (e.g., As, Cr, Tl). As a result, vernadite acts as a sink for many trace metal elements. In the environment, vernadite is often found associated with tectomanganates (e.g., todorokite and cryptomelane) of which it is thought to be the precursor. The transformation mechanism is not yet fully understood however and the fate of metals initially contained in vernadite structure during this transformation is still debated. In the present work, the transformation of synthetic vernadite (δ-MnO_2_) to synthetic cryptomelane under conditions analogous to those prevailing in soils (dry state, room temperature and ambient pressure, in the dark) and over a time scale of ~10 years was monitored using high-energy X-ray scattering (with both Bragg-rod and pair distribution function formalisms) and transmission electron microscopy.

**Results:**

Migration of Mn^3+^ from layer to interlayer to release strains and their subsequent sorption above newly formed vacancy in a triple-corner sharing configuration initiate the reaction. Reaction proceeds with preferential growth to form needle-like crystals that subsequently aggregate. Finally, the resulting lath-shaped crystals stack, with *n* × 120° (*n* = 1 or 2) rotations between crystals. Resulting cryptomelane crystal sizes are ~50–150 nm in the **ab** plane and ~10–50 nm along c*, that is a tenfold increase compared to fresh samples.

**Conclusion:**

The presently observed transformation mechanism is analogous to that observed in other studies that used higher temperatures and (or) pressure, and resulting tectomanganate crystals have a number of morphological characteristics similar to natural ones. This pleads for the relevance of the proposed mechanism to environmental conditions.

**Electronic supplementary material:**

The online version of this article (doi:10.1186/s12932-015-0028-y) contains supplementary material, which is available to authorized users.

## Background

Vernadite (and δ-MnO_2_, its synthetic analogue) is a nanocrystalline turbostratic birnessite, a phyllomanganate whose layers are built of (MnO_6_)^8−^ octahedra connected through their edges and separated by hydrated interlayer cations. Vernadite is ubiquitous in the environment, and probably results mainly from the aqueous oxidation of Mn^2+^ by bacteria [1–3], fungi [4–6], and higher living forms [7], as abiotic oxidation catalyzed by mineral surfaces is about two orders of magnitude slower [8–11]. Vernadite layers frequently contain vacancies and (or) isomorphic substitutions (substitution of layer Mn^4+^ by a foreign cation, e.g., Co^3+^, Mn^3+^, or Ni^2+^ [12–14]), both types of defects inducing a layer charge deficit. For example, layer charge was 0.86–1.22 and 1.58 valence unit (v.u.) per layer octahedron for samples produced by fungal strains [4] and by grass roots [7], respectively. Layer charge induces a high chemical reactivity, which is reinforced by the nanometric size of vernadite (typically 5–50 nm in the layer plane—[1, 15, 16]) and the induced proportion of reactive edge sites [3, 17, 18]. Vernadite also presents a high affinity for many trace elements such as transition metals (e.g., Co, Ni, Zn), actinides and rare earth elements [14, 19–36]. For example, vernadite is the main sink for Ni in mixed mineral/biotic systems (vernadite and *Pseudomonas Putida* biofilm [37, 38]). Vernadite can also oxidize organic pollutants and redox-sensitive elements such as arsenic [39–42], chromium [39–43], and thallium [34, 44], possibly because of the common coexistence of heterovalent Mn cations in its structure. Specifically, Mn^3+^ cations can be present both within octahedral layers and (or) adsorbed above layer vacancies, forming triple-corner-sharing complexes (^TC^Mn^3+^; Fig. 1 in [45]).

Additional interest in understanding vernadite structure arises from its frequent association with tectomanganates (i.e., tunnel structures) in the environment. Tectomanganates include a variety of minerals with [*n* × *m*] tunnel sizes, where *n* stands for the number of octahedra connected to form the “walls” of the tunnels, whereas *m* stands for the number of octahedra forming the “ceiling” and the “floor”. For example, vernadite is frequently found with todorokite and cryptomelane, [3 × 3] and [2 × 2] tunnel structures, respectively [26, 46–48]. Tectomanganates can form from phyllomanganate precursors [46, 49, 50] provided that they possess a particular crystal-chemistry, for example possess Mn^3+^ [51, 52]. Phyllomanganate-to-tectomanganate reaction mechanisms have been widely investigated owing to the tectomanganate potential as octahedral molecular sieves. Reaction products must have homogenous tunnel size for this purpose to achieve optimal efficiency and the relation between the layered precursor and the resulting tunnel structure is studied with special care (e.g., [49] and references therein and [53, 54]). Relations between layer and tunnel structures is also of interest in natural settings, mainly because defective todorokite (i.e., having mainly [3 × 3] tunnel size, together with [3 × *m*] size, *m* varying from 2 to 5) is found associated with vernadite in oceanic ferromanganese nodules. These nodules consist of alternating layers of iron and manganese oxides [31, 34, 55], the latter containing typically over 1 wt% of Ni [56–58], and are increasingly considered for their potential as a source of strategic trace metals including rare-earth elements. A comprehensive understanding of phyllomanganate to tectomanganate transformation, at the atomic scale, thus appears key to an improved prediction and modeling of the impact of structure defects (layer vacancies and isomorphic substitutions) on the fate of trace elements.

Observations of vernadite-to-todorokite transformation in natural samples are scarce, but suggest a topotactic reaction [46]. It was first reported by Golden and coworkers [59] for synthetic analogues and has since been repeatedly described for a variety of hydrothermal protocols. Recently, these protocols were reviewed by Atkins and coworkers [60], who also characterized the structural mechanisms of the δ-MnO_2_ to todorokite transformation at 100 °C and atmospheric pressure. These authors described the transformation as a four-stage process, starting with the formation of todorokite tunnel walls via layer kinking, followed by the growth of the particles along the [010] direction (i.e., along the tunnel direction) during a dissolution–recrystallization step and subsequent oriented attachment of the resulting particles along the [100] direction to form todorokite laths which then stack. A last step is described as the growth of todorokite crystals by Ostwald ripening. These authors mentioned also that the density of structural Mn^3+^ has to be “significant” to allow for a complete transformation and linked this condition to the ability of Mn^3+^ to induce layer kinking, owing to its Jahn–Teller distorted environment. Another important parameter appears to be the presence of an interlayer cation enforcing a ~10 Å layer-to-layer distance (e.g., Mg^2+^).

Similar to todorokite, cryptomelane formation in environmental conditions is little documented. A recent study [49] focused on δ-MnO_2_ samples equilibrated at pH values ranging from 3 to 10. Initially, all samples had a similar number of layer Mn^3+^ per octahedron (0.10 ± 0.02–0.14 ± 0.02), whereas the number of interlayer ^TC^Mn^3+^ increased with decreasing equilibration pH. Although such evolution might seem counter-intuitive because, by analogy with clay minerals, decreasing pH should increase proton competition for sorption [61–63], it is made possible by the fact that pH decrease is accompanied by partial layer dissolution and thus by an increase of Mn concentration in the equilibrium solution, making it possible for dissolved Mn to adsorb above vacancies [64]. Structural formulae from samples initially equilibrated at pH 3 and 10 were Na_0.06_^+^(H_2_O)_0.30_Mn_0.185_^3+^[Mn_0.12_^3+^Mn_0.71_^4+^Vac_0.17_O_2_] and Na_0.27_^+^(H_2_O)_0.30_Mn_0.095_^3+^[Mn_0.10_^3+^Mn_0.76_^4+^Vac_0.14_O_2_], respectively. In these formulae, species within brackets form the layer, those to the left the interlayer, Vac stands for layer vacancies, and all interlayer Mn sites (TC and triple-edge sharing [45] configurations) are summed. Samples were then dried, and aged in the dark. With time, it was observed that layer Mn^3+^ leaves the layer to form ^TC^Mn^3+^ above the newly generated vacancy. Partial transformation to cryptomelane was observed only for pH 3 sample that had the highest initial number of Mn^3+^ (layer plus interlayer). Samples initially equilibrated at pH 4–10 did not show evidence for transformation to cryptomelane, but had contrasting crystal chemistry (Na^+^_0.12_(H_2_O)_0.30_Mn_0.315_^3+^[Mn_0.74_^4+^Vac_0.26_O_2_] and Na^+^_0.27_(H_2_O)_0.30_Mn_0.205_^3+^[Mn_0.79_^4+^Vac_0.21_O_2_], respectively) after 8 years of ageing. The structure of pH 3 sample was not precisely determined, but crystals that transformed certainly had ~1/3 of ^TC^Mn^3+^ per layer octahedron [49]. Still, many questions remain open as to the transformation mechanisms. In particular, it was unclear if the transformation was homogeneous at the crystal scale (if some crystals transformed to cryptomelane while others remained lamellar) or if it only affected portions of the crystals (topotactic transformation). Finally, tectomanganates observed in natural environments are larger than typical vernadite [65, 66]. The mechanisms leading to crystal growth during or following vernadite to cryptomelane transformation (e.g., dissolution/recrystallization, aggregation, oriented attachment, Ostwald ripening) could not be elucidated.

The present study focuses further on the structure of aged δ-MnO_2_ samples studied by Grangeon and coworkers [49]. Samples that were initially equilibrated at pH values of 3, 4, 8, and 10, and then left ageing in the dark in the dry state for 10 years are hereafter referred to as MndBiXX_10y, where XX stands for the equilibration pH. These samples were selected because of their contrasting structures after 8 years of ageing (i.e., 2 years before the present study). MndBi3_10y and MndBi10_10y respectively represent the highest degree of transformation and an aged δ-MnO_2_ that retained its original lamellar structure. In the present study, synchrotron X-ray diffraction data is analyzed with both Bragg-rod and pair distribution function (PDF) methods to determine the sample structure as a function of pH, after two more years of ageing. Bragg-rod formalism is used to determine whether samples were partially transformed to cryptomelane, and to determine the size of coherent scattering domains (CSD) and the number of ^TC^Mn^3+^. PDF analysis is used to determine if layer vacancies are ordered and to cross-check the number of ^TC^Mn^3+^. In addition, transmission electron microscopy (TEM) will be used to determine actual crystal sizes and to provide textural and structural constraints on the vernadite-to-cryptomelane transformation mechanisms. Scanning TEM will be used to reveal structural heterogeneities at the atomic scale.

## Results

### Analysis of X-ray diffraction patterns and Bragg-rod modeling

**Fig. 1:**
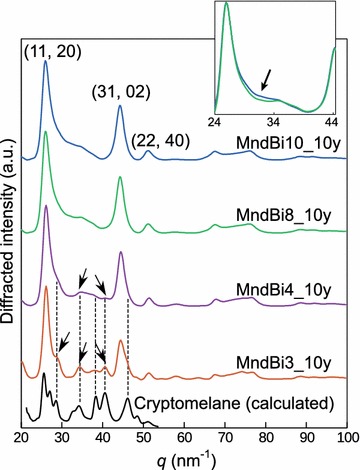
XRD patterns of the studied samples and of a calculated cryptomelane pattern. *Main panel*, from *top* to *bottom* XRD patterns of MndBi10_10y, MndBi8_10y, MndBi4_10y, MndBi3_10y and a calculated cryptomelane pattern (over the 21–53 nm^−1^ interval, CSD size of 6 nm). Patterns are scaled for clarity, as maximum intensity diffracted by δ-MnO_2_ is about 3 % of the maximum intensity diffracted by cryptomelane. *Arrows* point out to main modulations attributable to a cryptomelane-like structure in MndBi3_10y and MndBi4_10y patterns. MndBi10_10y and MndBi8_10y patterns are overlaid in the *inset* at the *top right* to highlight that the hump at 30–33 nm^−1^ is better defined in MndBi8_10y

X-ray diffraction (XRD) patterns can be divided in two groups (Fig. 1). Patterns of MndBi8_10y and MndBi10_10y are typical for δ-MnO_2_, exhibiting reflections at 25.9 nm^−1^ (2.43 Å), 44.3 nm^−1^ (1.42 Å), and 51.3 nm^−1^ (1.22 Å) that can be assigned to [11, 20], [31, 02], and [22, 40] bands, using a C-centered unit cell with *γ* = 90° [67, 68]. Compared to MndBi10_10y, the hump at 30–33 nm^−1^ (2.1–1.9 Å) is better defined in MndBi8_10y (Fig. 1), likely as the result of a higher density of heavy interlayer species at TC sites. The symmetry and position of their [31, 02] bands suggest that MndBi10_10y and MndBi8_10y both have hexagonal layer symmetry [67] and similar *a* and *b* unit-cell parameters [69]. CSD sizes along the c* axis and layer-to-layer distance could not be determined owing to present experimental conditions that prevented modeling of low-angle 00*l* reflections.

Compared to MndBi8_10y and MndBi10_10y, additional reflections are observed at 29.0 nm^−1^ (2.17 Å), 34.4 nm^−1^ (1.83 Å), and 40.6 nm^−1^ (1.55 Å) in MndBi3_10y and MndBi4_10y. These reflections are more intense in MndBi3_10y than in MndBi4_10y (Fig. 1) and can be attributed to a cryptomelane-like structure. MndBi3_10y and MndBi4_10y are thus well-suited to study initial stages of the phyllomanganate-to-tectomanganate transformation, with part of MndBi3_10y and, to a lesser extent, of MndBi4_10y crystals having a cryptomelane-like structure.

**Fig. 2:**
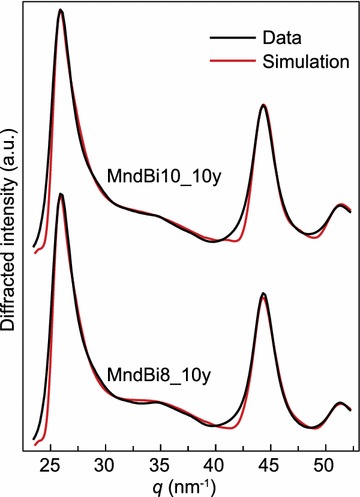
Experimental and calculated XRD patterns of MndBi10_10y and MndBi8_10y. Experimental (*black solid line*) and calculated (*red solid line*) XRD patterns of MndBi10_10y (*top*) and MndBi8_10y (*bottom*). Structure parameters are available in Table 1

Quantitative modeling of *hk* bands from MndBi8_10y and MndBi10_10y XRD patterns is shown in Fig. 2. Modeling of MndBi3_10y and MndBi4_10y patterns was not undertaken owing to the presence of cryptomelane, whose most intense reflections overlap δ-MnO_2_ [11, 20] band (Fig. 1). Relative intensity ratios calculated for cryptomelane and δ-MnO_2_ having similar CSD sizes (~6 nm) indicate that cryptomelane represent <5 % of the crystalline phases in the sample. MndBi10_10y has a CSD size in the **ab** plane of 5.8 nm and contains 0.11(1) ^TC^Mn^3+^ per layer octahedron (Table 1), lower than the value obtained 2 years sooner [0.165(10) per layer octahedron]. Similar decrease of ^TC^Mn^3+^ with time was previously observed [70] and attributed to ^TC^Mn^3+^ to Mn^4+^ oxidation with time, followed by migration to the layer. This phenomenon may also be at play in other samples but could not be identified either because the structures were not refined (MndBi3_10y and MndBi4_10y) or because the structure of the same sample was not determined 2 years sooner owing to a restricted amount of sample available for analysis with conventional XRD instruments (MndBi8_10y). Finally, the number of interlayer H_2_O molecules in MndBi10_10y was refined to 0.12 per interlayer site, slightly more than 2 years sooner (0.10). MndBi8_10y has CSD size in the **ab** plane of 6.2 nm, contains 0.02 more ^TC^Mn^3+^ and 0.02 more layer vacancies per layer octahedron than MndBi10_10y.

**Table 1:** Main structural parameters extracted from analysis of high energy X-ray scattering data, in the PDF and Bragg-rod approaches

Sample	^TC^Mn (per layer octahedron)		*b* (Å)^a^		*c* (Å)
	From PDF	From Bragg-rod	From PDF	From Bragg-rod	From PDF
MndBi3_10y	0.28(1)	n.d.	2.854(1)	n.d.	7.13(1)
MndBi4_10y	0.19(2)	n.d.	2.853(4)	n.d.	7.15(3)
MndBi8_10y	0.12(2)	0.13 (1)	2.868(4)	2.855	7.16(3)
MndBi10_10y	0.09(2)	0.11 (1)	2.869(4)	2.855	7.18(3)

Anisotropic Debye–Waller factors, as well as other fit parameters, determined from PDF analysis, are available in Additional file 1: Data S1. Uncertainties on results from PDF simulation is indicated under brackets, and represents the uncertainty on the value of the last digit

^a^ *a* was constrained to be equal to √3 *b*

### Analysis of PDF data

As PDF analysis will hereafter focus on short-range order, MndBi3_10y and MndBi4_10y, which contain a minor amount of cryptomelane, will be treated as pure δ-MnO_2_ owing to the structural similarities between the two species. Indeed, Mn–Mn pairs from δ-MnO_2_ layers are similar to those in cryptomelane walls or floor/ceiling, and pairs formed by layer Mn and ^TC^Mn in δ-MnO_2_ are similar to those formed by Mn atoms from adjacent walls and floor/ceiling in cryptomelane. An implication of this similarity is that PDF data cannot be used, in the present study, to detect a minor amount of cryptomelane in the samples.

**Fig. 3:**
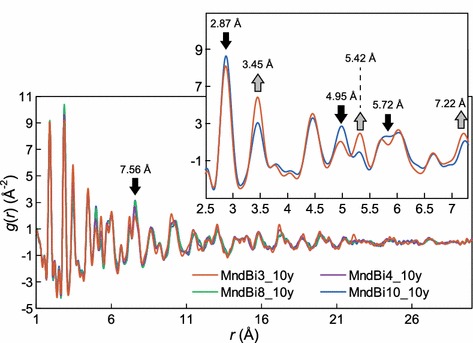
Comparison of PDF of all studied samples. *Main panel* PDF of MndBi3_10y (*orange*), MndBi4_10y (*purple*), MndBi8_10y (*green*) and MndBi10_10y (*blue*). *Inset* at the *top right* shows alternations of decreasing (*black arrows*) and increasing (*grey arrows*) correlations, from MndBi10_10y to MndBi3_10y. Only MndBi10_10y and MndBi3_10y are shown in the *inset* to ease visualization

**Fig. 4:**
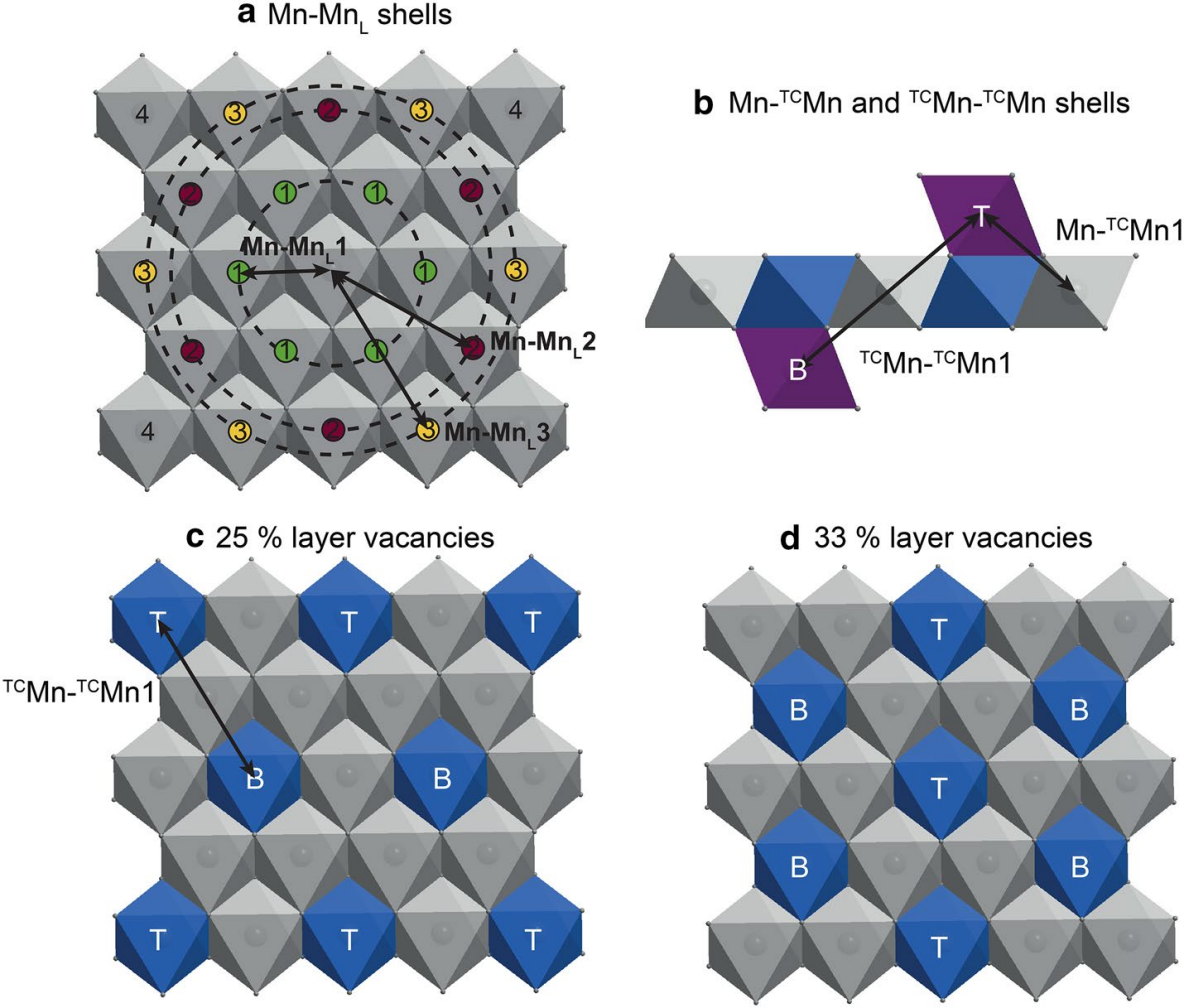
Scheme of vernadite layer and of atomic pairs involving Mn atoms. **a** Scheme of a vernadite layer, seen along c* with *grey* octahedra representing (MnO_6_)^8−^ octahedra. The first three atomic shells formed by layer Mn around a given layer Mn (Mn–Mn_L_X pairs, were X stands for the atomic shell) are highlighted with *concentric circles*. *Green full circles* with a “1” mark, *red full circles* with a “2” mark, *orange full circles* with a “3” mark, and “4” marks respectively schematize layer Mn belonging to the first, second, third and fourth (only partly represented) atomic shells. **b** Scheme of a layer seen perpendicular to c*. *Blue* octahedra are layer vacancies, which are capped on one side by ^TC^Mn^3+^ (*purple* octahedra). The first layer Mn to ^TC^Mn pair (Mn–^TC^Mn1) as well as the first ^TC^Mn to ^TC^Mn pair, when the two atoms are on opposite sides of the layer (^TC^Mn–^TC^Mn1), are represented. “T” stands for “top” and means that a ^TC^Mn is located on the other side of the layer as compared to a ^TC^Mn labeled “B” (standing for “bottom”). **c** Scheme of a layer containing 1/4 vacancy (*blue* octahedra) per layer octahedron, vacancies being ordered. *Purple* octahedra are omitted for clarity, only the captions “T” and “B” are left to materialize ^TC^Mn. The first ^TC^Mn–^TC^Mn pair is highlighted with *arrows*. **d** Same as **c**, but with a layer containing 1/3 vacancy per layer octahedron

From a qualitative examination of the PDF data (Fig. 3), a systematic evolution is observed with sample equilibration pH. From MndBi10_10y to MndBi3_10y, correlations at 2.87, 4.95, 5.72, and 7.56 Å decrease in intensity (although the third appears less affected). These correlations are attributed to atomic pairs involving two layer Mn atoms and forming the first, second, third, and fourth Mn shells around a given layer Mn (Mn–Mn_L_1, Mn–Mn_L_2, Mn–Mn_L_3 and Mn–Mn_L_4 shells—Fig. 4a). Contrastingly, correlations at 3.45 and 5.32 Å increase in intensity. They are attributed to pairs formed by a layer Mn atom and a ^TC^Mn (Mn–^TC^Mn pairs—Fig. 4b), with ^TC^Mn at vacancies belonging respectively to the first (Mn–^TC^Mn1) and second (Mn–^TC^Mn2) Mn–Mn_L_ shells (Fig. 4a, b, c). The number of layer vacancies thus increases from MndBi10_10y to MndBi3_10y, ^TC^Mn being sorbed above these vacancies. The first, second and fourth Mn–Mn_L_ shells are more affected than the third shell. All these shells contain, in a defect-free δ-MnO_2_ layer, 6 Mn atoms. Thus, a layer vacancy normally affects equally all of these shells, and the observed behavior can most straightforwardly be explained by an ordered layer vacancy distribution. Finally, the correlation at 7.22 Å increases in intensity from MndBi10_10y to MndBi3_10y. It corresponds to a ^TC^Mn–^TC^Mn pair, with ^TC^Mn atoms being on opposite sides of layer vacancies separated from each other by one layer Mn atom (^TC^Mn–^TC^Mn1 in Fig. 4c). All these correlations and in particular the ^TC^Mn–^TC^Mn one are typical for δ-MnO_2_ layers having 0.25 layer vacancy per layer octahedron and ^TC^Mn as in Fig. 4c. In addition, the correlation at 6.13 Å (Mn–^TC^Mn3 pair, i.e., ^TC^Mn above a vacancy belonging to the Mn–Mn_L_3 shell) may indicate the minor presence of domains with 0.33 vacancy per layer octahedron in low pH samples (Fig. 4d). Such domains could correspond to the cryptomelane-like structure or to their precursors.

**Fig. 5:**
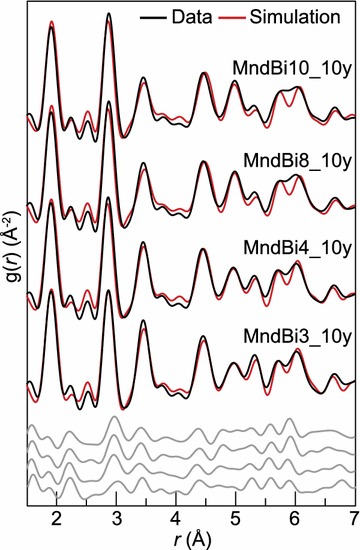
Experimental and calculated PDF of all studied samples. From *top to bottom* experimental (*black solid line*) and calculated (*red solid line*) PDF of MndBi10_10y, MndBi8_10y, MndBi4_10y and MndBi3_10y, and difference plots (*solid grey line*). Fit interval was 1.5–7 Å. R_w_ was respectively 25.8, 25.4, 24.0 and 20.8 %

As discussed hereafter and by Manceau and coworkers [70], δ-MnO_2_ PDF data are affected by sample turbostratism, that is by the systematic presence of random translation and/or rotation between successive layers. Turbostratism decreases the intensity of correlations for *r* values higher than the layer-to-layer distance, because coherency between adjacent layers is lost. In the present samples both ~7.2 and ~10 Å layer-to-layer distances were observed (see below), the former being by far the most frequent. As a consequence, refinement was restricted to the 1.5–7 Å intra-layer interval which is not affected by turbostratism. The model from Manceau and coworkers [70] was used and no attempt was made to refine atomic coordinates nor layer symmetry. Refined parameters corresponding to the simulations shown in Fig. 5 are reported in Table 1 and Additional file 1: Data S1. With decreasing pH, the abundance of ^TC^Mn^3+^ steadily increased, from 0.09(2) per layer octahedron in MndBi10_10y to 0.28(1) per layer octahedron in MndBi3_10y (Table 1), consistent with previous studies [49, 70, 71]. Layer parameters decreased from *b* = 2.869(4) Å in MndBi10_10y to *b* = 2.854(1) Å in MndBi3_10y, certainly as a result of the increase in ^TC^Mn^3+^ [49]. These values are systematically lower [0.013(4)–0.014(4) Å] than those obtained using the Bragg-rod approach (Table 1) possibly as a consequence of layer bending [70].

### Morphological and structural evolution with pH as seen by transmission electron microscopy

Bragg-rod and PDF data analysis allowed probing structure of coherent scattering domains but could not determine their distribution within crystals, and in particular the possible coexistence of δ-MnO_2_ and cryptomelane domains within crystals. As they are representative of samples undergoing the maximum and minimum degree of structural conversion, MndBi3_10y and MndBi10_10y were investigated by TEM to gain further insights into transformation mechanisms.

**Fig. 6:**
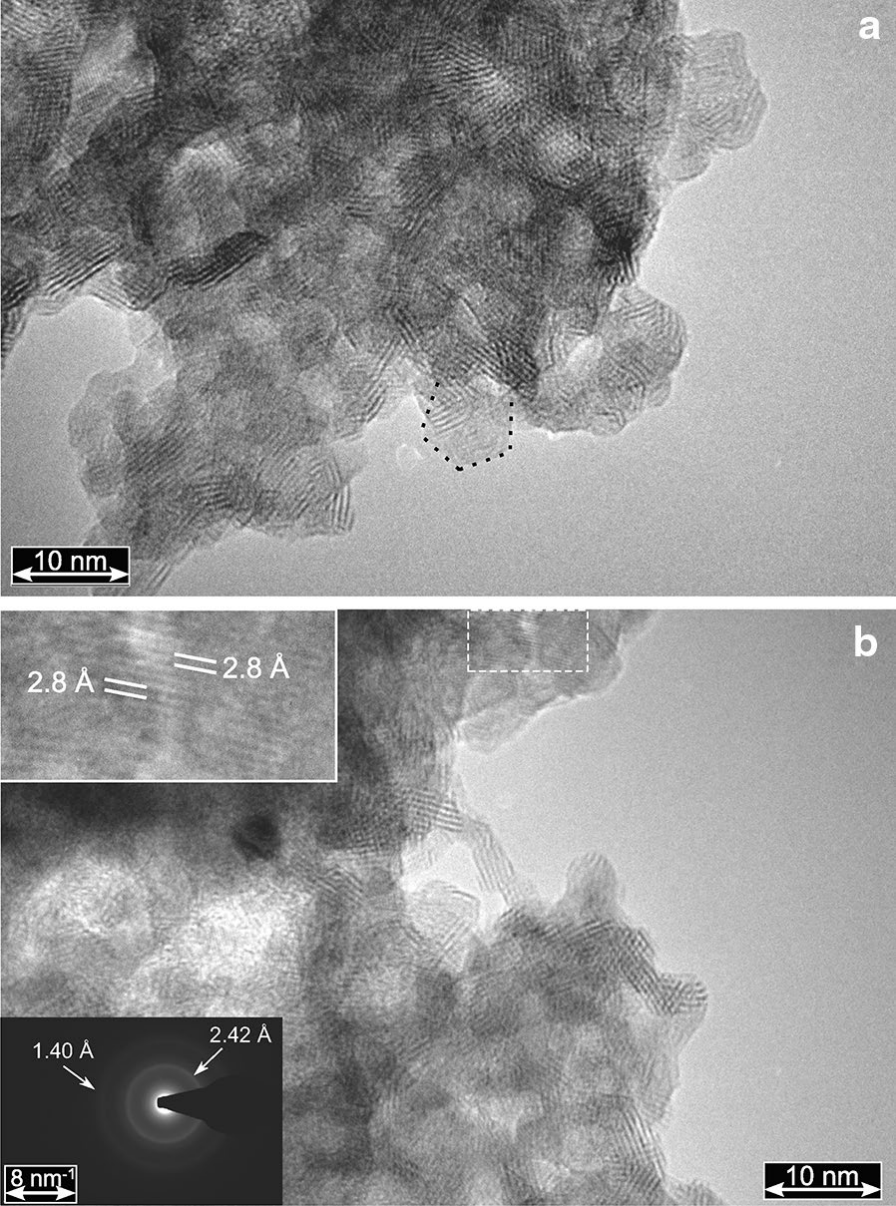
TEM observation of MndBi10_10y. **a** Typical crystal size in the **ab** plane was 5–20 nm, and some crystals had pseudo-hexagonal shape (highlighted with a *black dotted line*). **b** *Main panel* is a view from other crystals from MndBi10_10y. *Inset* at *top left* is a magnified view of the zone marked with *white dotted line*. It shows interference fringes separated by 2.8 Å, typical for δ-MnO_2_ *b* lattice parameter. SAED pattern collected in this area (*bottom left*) shows only weak maxima at 2.42 and 1.40 Å, typical for turbostratic phyllomanganates

MndBi10_10y was composed of crystals having homogenous size and morphology, often found aggregated in a xerogel-like configuration. Selected area electron diffraction (SAED) patterns were typical for δ-MnO_2_, with two broad diffraction maxima at ~2.42 and ~1.40 Å (Fig. 6) that correspond to [11, 20] and [31, 02] diffraction bands. Crystal sizes in the **ab** plane range 5–10 nm, consistent with CSD size determined from XRD pattern modeling (5.8 nm). Some crystals exhibited hexagonal shape in the **ab** plane (Fig. 6a), as observed in other δ-MnO_2_ samples [45, 70]. Finally, interference fringes having spacing of ~2.8 Å could be observed (Fig. 6b) and are attributed to the *b* parameter.

**Fig. 7:**
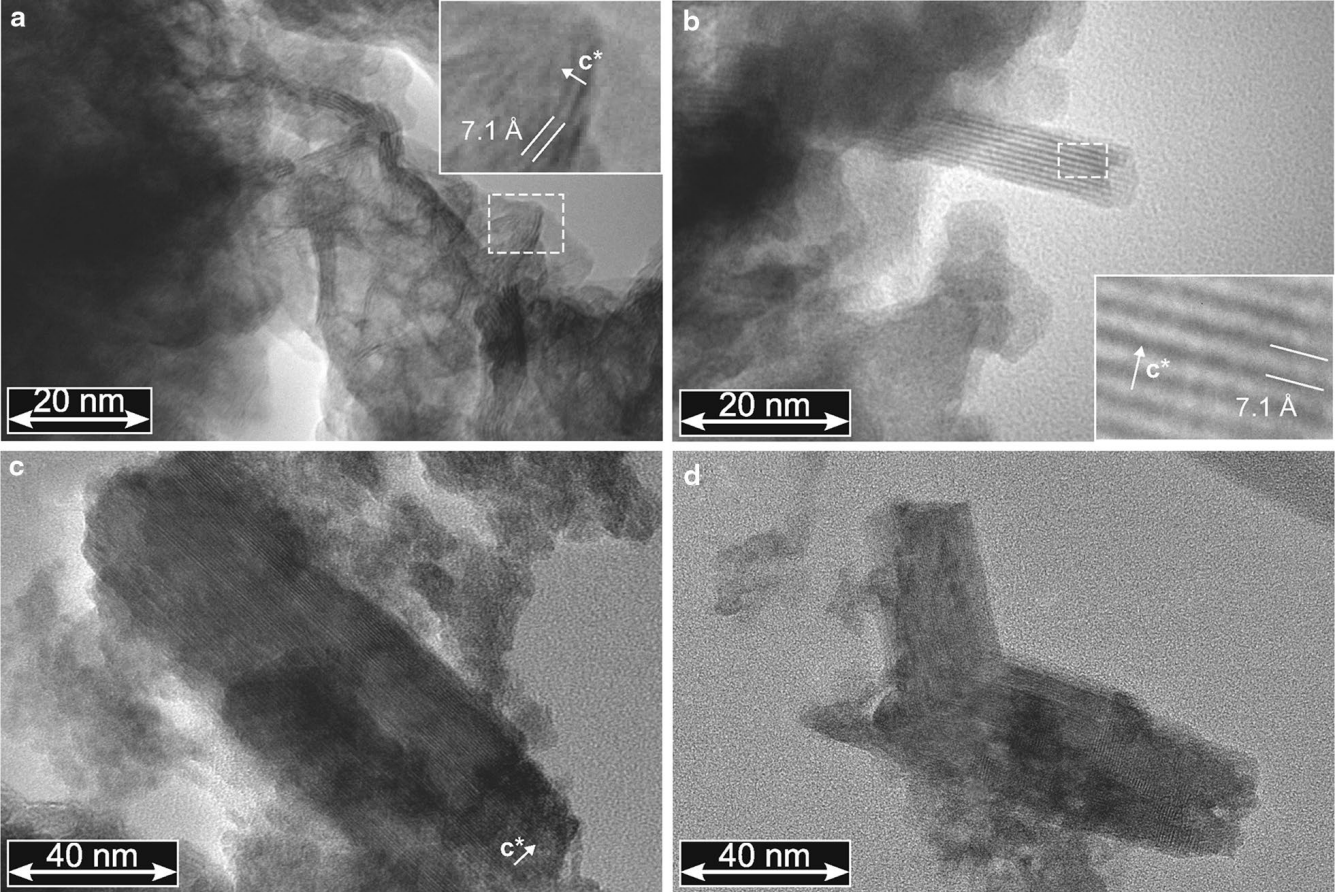
The four different types of crystals observed in MndBi3_10y. Examples of TEM observations made on the four different types of crystals observed in sample MndBi3_10y. Crystal type 1 had a xerogel-like configuration and typical crystal size of 5–20 nm (**a**), crystal type 2 had needle-like shape and an extension of 10–30 nm (**b**), crystal type 3 had lath-like shape and typical size of about 20–150 nm (**c**), and crystal type 4 was built of type 3 crystals with rotation of *n* × 120° (*n* = 1 or 2) between adjacent layers (**d**)

**Fig. 8:**
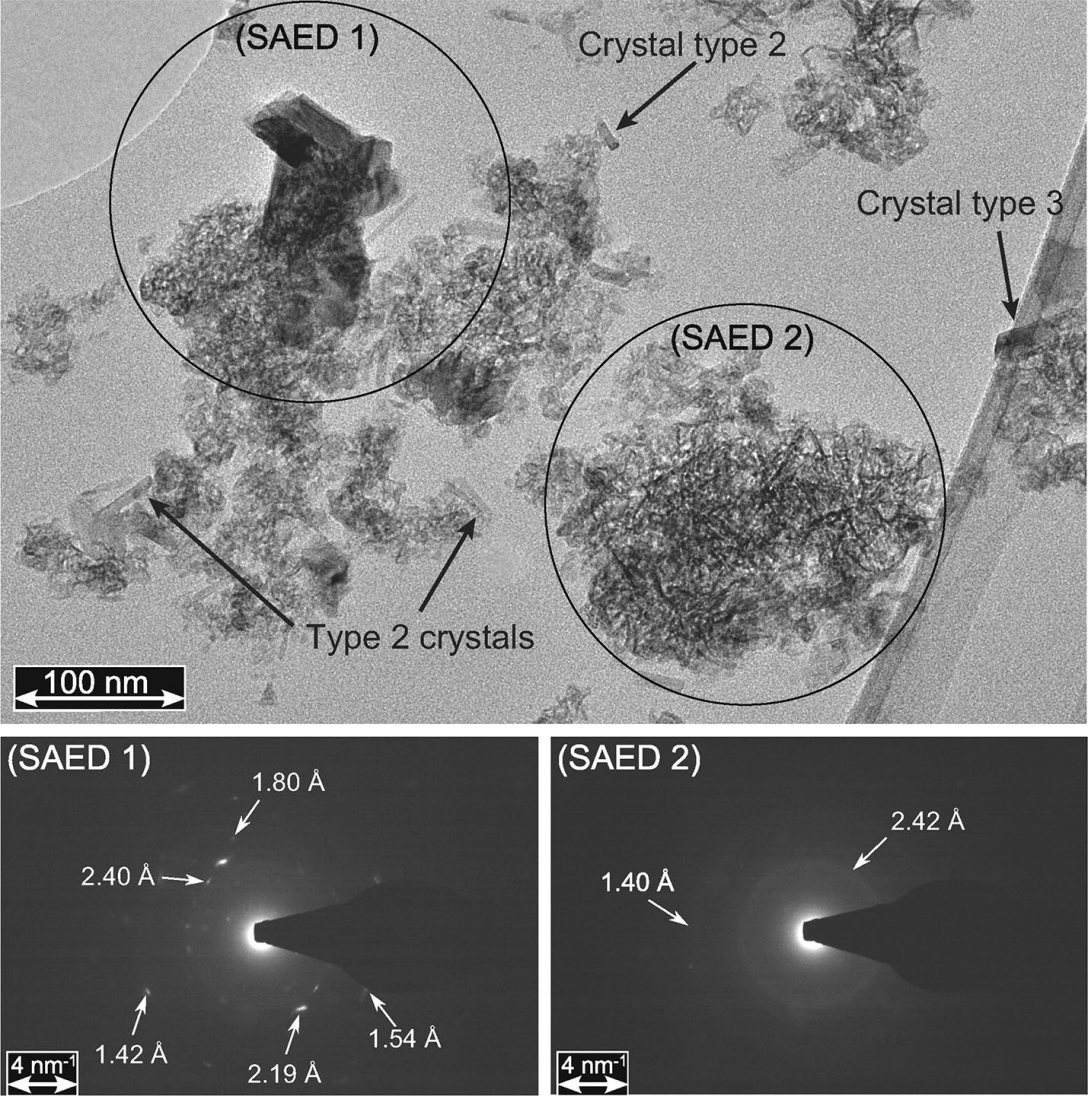
TEM observation of MndBi3_10y and SAED patterns of type 1 and type 4 crystals. *Top panel* Low-magnification view of crystals from MndBi3_10y. *Circles* indicate areas which were subjected to electron diffraction. *Circle* at the *top left* (SAED 1) targeted crystal type 4, which had a diffraction pattern exhibiting features characteristics for cryptomelane, and in particular *d*-spacing of 2.40, 2.19, 1.80, 1.54 and 1.42 Å (pattern at the *bottom left*). *Circle* at the *bottom right* (SAED 2) targeted crystal type 1 and had a signal typical for δ-MnO_2_, with distances of 2.42 and 1.40 Å (pattern at the *bottom right*). Crystals types 2 and 3 which are most easily distinguishable are pointed out with *arrows*

**Fig. 9:**
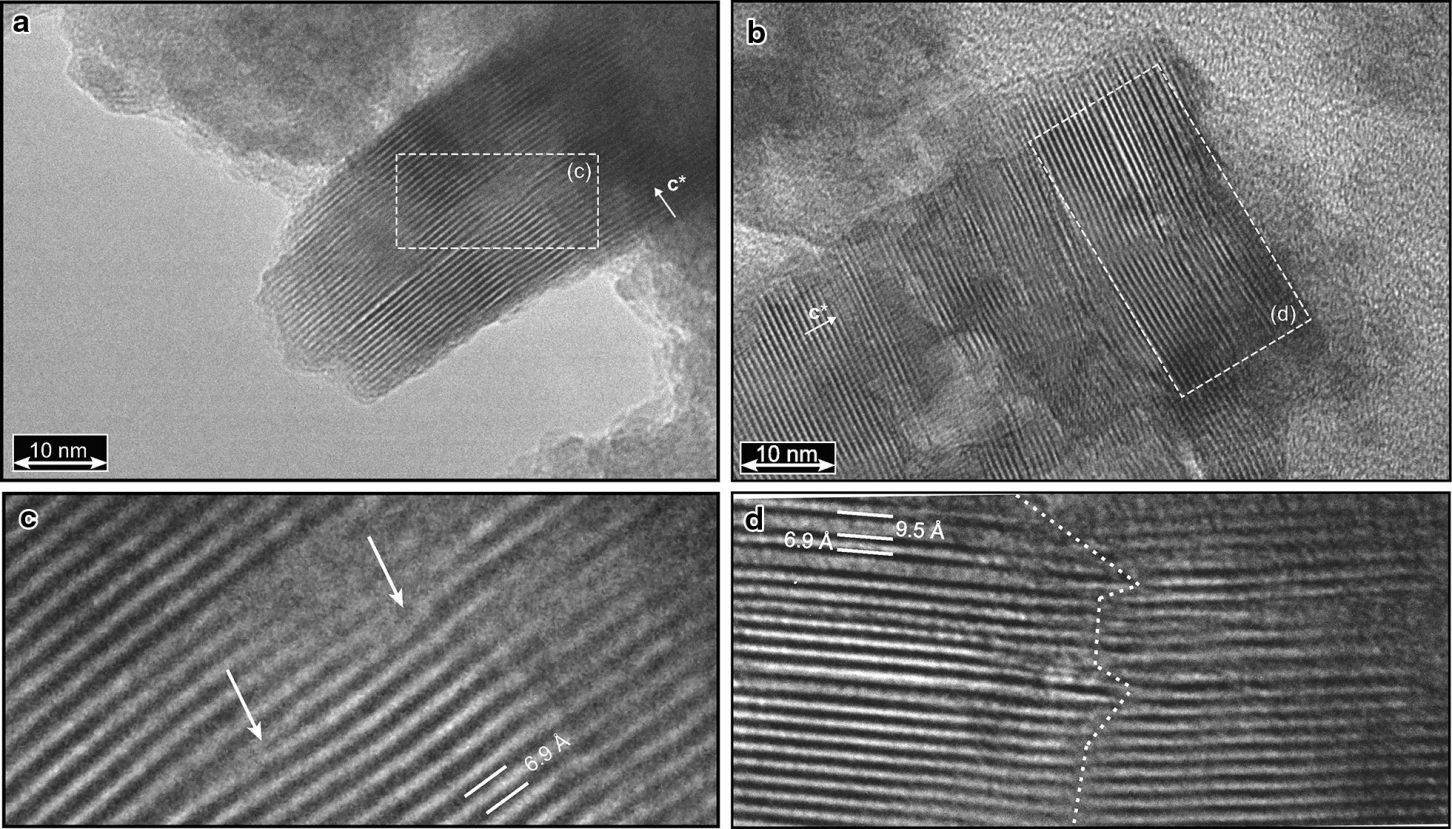
TEM observations of two different type 3 crystals from MndBi3_10y. **a** Shows the presence of a dislocation perpendicular to c*, highlighted with *arrows* in **c** which corresponds to the area delimited with a *dotted line* in **a**. **b** Shows evidence for the disruption of lattice fringes perpendicular to **ab** and thus certainly witnesses aggregation of two crystals, as highlighted with a *dotted line* in **d** which is a magnified view of the area delimited with a *dotted line* in **b**. In both crystals presented here, lattice fringes separated by 6.9 and 9.5 Å could be observed, close to the values expected for the layer-to-layer distances in δ-MnO_2_ having respectively one and two planes of interlayer water molecules

Observation of MndBi3_10y samples revealed a much more complex assemblage. The sample contained at least four types of crystals distinguishable on the basis of their morphology and size (Figs. 7, 8). Crystal type 1 had sizes of 5–20 nm, similar to fresh samples (data not shown) and MndBi10_10y. Crystal type 2 had a needle-like morphology, typically 10–30 nm in length. Crystal type 3 had lath-like morphology, with its long distance in the **ab** plane (20–150 nm) up to ~5–10 times larger than crystal type 2. It was also typically 2–10 times wider, and interference fringes were frequently disrupted, as if it was built of aggregated type 2 crystals (Fig. 9). Aggregation was observed to occur within the **ab** plane, in agreement with the aggregation mechanism proposed previously [50, 60, 72]. It was also observed along c*, with disruption of interference fringes that had geometrical shapes identical to previous observations [60, 72, 73] and indicate that growth may also take place through stacking along c* of two or more type 2 crystals. Finally, crystal type 4 often had typical sizes of 50–150 nm in the **ab** plane and 10–50 nm along c*, and was built of stacks of lath-like layers resembling type 3 crystals rotated by *n* × 120° (*n* being equal to 1 or 2) relative to each other.

**Fig. 10:**
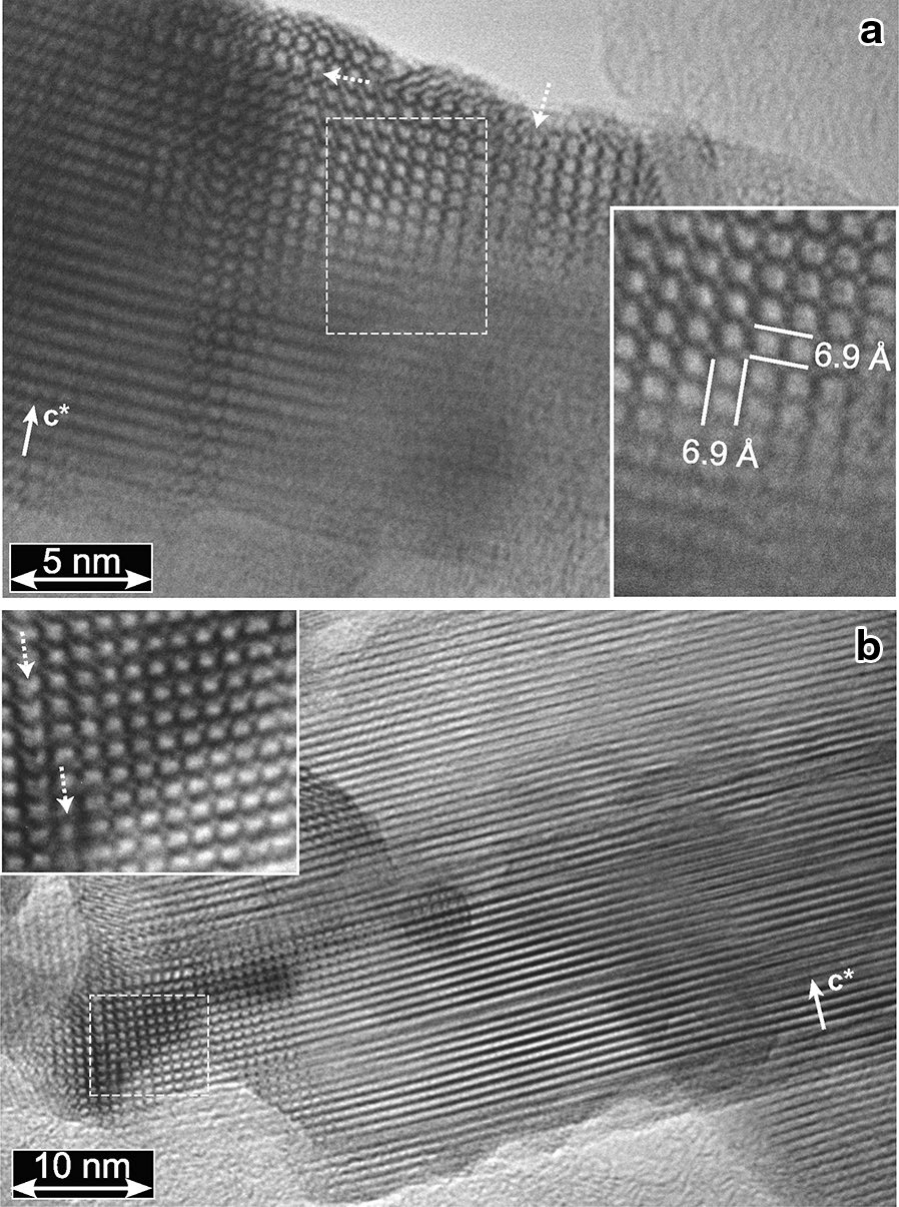
TEM imaging of cryptomelane domains in type 4 crystals of MndBi3_10y. **a**, **b** TEM observations of two different crystals type 4 from sample MndBi3_10y. In both crystals, a tunnel-like structure is observed. It mainly has a tunnel size of 6.9 Å × 6.9 Å, but structures having heterogeneous tunnel size are also observed (*arrow with dotted line*). Areas with *dotted lines* indicate zones corresponding to the enlarged pictures (*bottom right* of **a** and *top left* of **b**)

Similar to MndBi10_10y, crystal type 1 in MndBi3_10y exhibited SAED patterns typical for δ-MnO_2_, with only weak diffraction maxima at 2.42 and 1.40 Å (Fig. 8). Contrastingly, crystal type 4 exhibited additional reflections (e.g., at 2.19, 1.80, and 1.54 Å—Fig. 8) attributed to cryptomelane. Consequently, MndBi3_10y is built of four main types of crystals: crystal type 1 is a phyllomanganate, whereas crystal type 4 locally has a tectomanganate-like structure. We propose that crystals type 2 and 3, that could not be investigated by SAED as no isolated crystal or homogeneous aggregate could be found, have an intermediate structure. When viewed perpendicular to the c* axis, crystal type 4 systematically showed the presence of tunnel-like structures, with sizes of 6.9 Å × 6.9 Å typical for cryptomelane (Fig. 10). The minor presence of heterogeneous tunnel size (dotted arrows in Fig. 10) is likely. Finally, analysis of images collected on type 1, 2 and 3 crystals (Figs. 7a, b, 9) reveal distances of ~6.9–7.1 Å, consistent with layer-to-layer distance of phyllomanganates hosting a single plane of interlayer H_2_O molecules. 9.5 Å distances expected for phyllomanganates hosting two planes of water molecules [74] were also observed, although much less frequent. This uncommon persistence of highly hydrated states under TEM vacuum conditions [60, 75] is likely related to sample impregnation in resin prior to analysis.

**Fig. 11:**
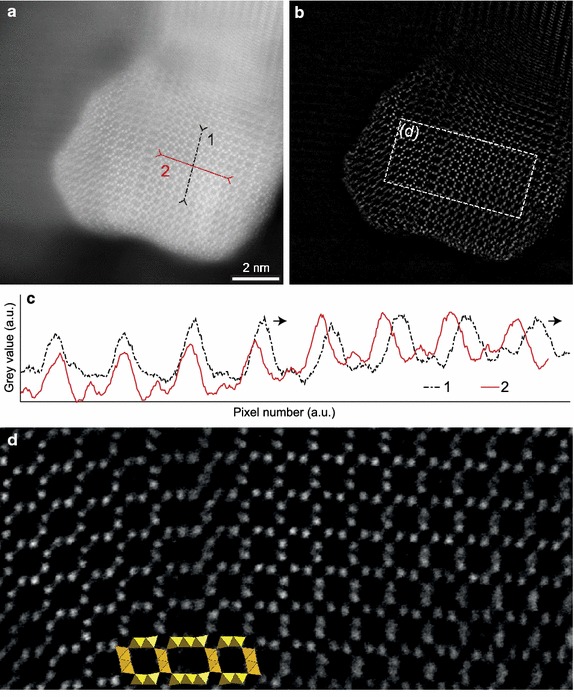
STEM imaging of a cryptomelane domain in crystal type 4 of MndBi3_10y. STEM observation of a cryptomelane domain in MndBi3_10y (**a**). Electron density is maximum when color is *white*, and minimum when color is *black*. **b** Is the same image after post-processing. Most of the particle is made of [2 × 2] tunnel structures, but [3 × 2] structures are also visible. **c** Shows the pixel *grey* values in two transects (delimited in **a**) along and perpendicular to tunnel walls. *Arrows* highlight the phase shift occurring between the two signals and thus the difference in tunnel size parallel and perpendicular to the tunnel walls. **d** Is a magnified view of part of **b** (area delimited with a *dotted line* in **b**), with a sketch of [2 × 2] and [2 × 3] tunnel structures overlaid to ease interpretation

Additional high-resolution STEM image was obtained at 100 keV (Fig. 11) to minimize structure evolution under the electron beam and to improve resolution in an effort to investigate the possible segregation of heterovalent Mn cations. Consistent with TEM observations (Fig. 10), [2 × 3] tunnels were observed in crystal type 4 together with prevailing [2 × 2] tunnels size, with individual Mn atoms being clearly observed. The latter tunnels have contrasting floor/ceiling and wall dimensions, with their small dimension (supposedly the former phyllomanganate layers) ~5 % smaller than the perpendicular dimension (Fig. 11c). This supports the hypothesis of Mn^3+^ atoms (that have a larger distorted coordination octahedron) segregation in the walls whereas Mn^4+^ atoms are mainly present in tunnel floors and ceilings. Finally, the structure was found to suffer from bending (Fig. 11d).

## Discussion

### Mechanism of the phyllomanganate to tectomanganate transformation

To our knowledge, this study is the first to document the transformation of vernadite to cryptomelane under conditions that can be considered relevant for soils, i.e. at room temperature, ~10^5^ Pa, under unsaturated conditions, and in the dark. As previously discussed [49], this structural transformation requires a locally high number of Mn^3+^ (~0.33 per layer octahedron) in the octahedral layer. This can be obtained experimentally by equilibrating phyllomanganates at low pH values (≤4) that can be observed in forest soils, soils developed on parent granite or gneiss, or organic-rich soils [76]. Such assumption is confirmed by independent laboratory experiments which show that δ-MnO_2_ to cryptomelane transformation is favored at low pH [72].

From many viewpoints, the studied transformation is similar to that of δ-MnO_2_ to todorokite described by Atkins and coworkers [60]. The contrasting reaction products obtained in the two studies are most likely due to the different nature of interlayer cation in the initial phyllomanganate (Na^+^ in the present study and Mg^2+^ for Atkins and coworkers). The first reaction step differs however as Atkins and coworkers propose that the presence of layer Mn^3+^ leads to layer kinking, owing to their Jahn–Teller distorted coordination sphere. In our opinion, reaction first step rather corresponds to the migration of Mn^3+^ from the layer to the interlayer, which allows also releasing strains related to the Jahn–Teller distortion of Mn^3+^ octahedra. This interpretation is consistent with the infrared data of Atkins and coworkers that showed an increased density of ^TC^Mn during the initial steps of the transformation. The proposed mechanism accounts also for their observation, corroborated by Feng and coworkers [53], that transformation occurs at constant mean Mn oxidation degree. In contrast to the hypothesis of Atkins and coworkers this initial step is likely not thermally triggered but reaction kinetics is enhanced with increasing temperature. The next steps of the reaction described by Atkins and coworkers are also observed in the present study. Needle-like crystal type 2 results indeed from the crystal growth along tectomanganate tunnels proposed by Atkins and coworkers, whereas lath-like crystal type 3 results from crystal growth perpendicular to tectomanganate tunnels (by coalescence of type 2 crystals along their long dimension). Finally, crystal type 4, built of lath-like units rotated by *n* × 120° (*n* being equal to 1 or 2), results from stacking of crystal type 3. The crystallographic axes along which crystal growth/aggregation takes place could not be determined in the present study, as SAED patterns could not be collected on type 2 and 3 crystals, and as instrumental limitations hampered the observations of in-plane lattice fringes. A last transformation step was described by Atkins and coworkers as Ostwald ripening and would involve cryptomelane crystal growth. This final step could not be observed in the present samples as evidence for a cryptomelane-like signal could only be found in crystal type 4 (Fig. 8). To date, only Bodeï and coworkers [46] have investigated in detail the mechanisms of phyllomanganate (vernadite) to tectomanganate (todorokite) transformation in natural samples. Collation of the present study with that of these authors is hampered however by numerous unknowns inherent to natural systems, such as the density of layer and interlayer Mn^3+^ in the initial phyllomanganate and the structural variety of natural tectomanganates [77, 78]. However, several clues suggest that the observed transformation is similar to that occurring in natural systems. First, proposed transformation mechanisms are valid both in surface soil conditions (present study) and in saturated conditions [50, 72], making them relevant to a variety of natural systems. Second, naturally occurring tectomanganates frequently exhibit textures similar to those observed in experimental studies (Figs. 7, 8; [51, 53, 60, 79, 80]) with rotations of aggregated crystals by *n* × 120° [46, 66, 79]. Third, transformations occur via topotactic transformation in both experimental and natural systems and are heterogeneous at the crystal scale. In particular, transformation systematically affects only part of the crystals and the transformed parts show heterogeneous tunnel sizes. This latter point is certainly related to an imperfect distribution of Mn^3+^ atoms (^TC^Mn^3+^ + layer Mn^3+^) in the initial phyllomanganate structure.

## Conclusion

**Fig. 12:**
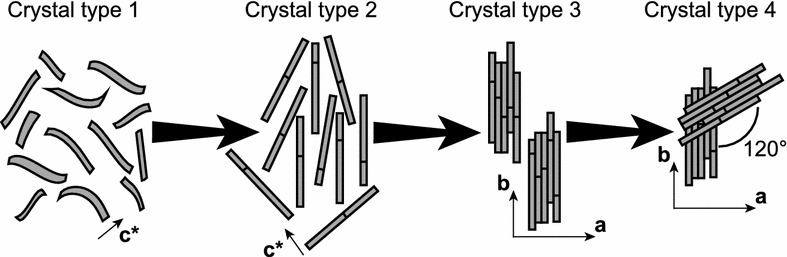
Proposed morphological and structural evolution with time for sample MndBi3_10y. From *left to right*, structure evolution starts from rumpled nano-sheets of phyllomanganates, proceeds via aggregation and oriented attachment to form laths which then stack along c*, with rotations of ±120° between adjacent laths, to form crystals which contain tectomanganate domains. Assignment of *a* and *b* directions was performed according to Atkins and coworkers [60], as such information could not be obtained in the present study

The phyllomanganate-to-tectomanganate transformation (Fig. 12) was studied using a combination of high-energy X-ray scattering and transmission electron microscopy. It was confirmed that this transformation is triggered when the number of ^TC^Mn^3+^ locally reaches ~0.33 per layer octahedron. The possible influence of Mn^4+^ to Mn^3+^ photo-reduction [81] on the transformation remains to be investigated. Part of ^TC^Mn^3+^ cations result from the migration of layer Mn^3+^ to the interlayer, which demonstrates that prediction of vernadite to tectomanganate transformation requires a sound understanding of vernadite structure, including quantification of both ^TC^Mn^3+^ and layer Mn^3+^ in starting material. In addition, the nature of the tectomanganate that will be formed is dependent on the nature of the interlayer cation: if the cation is capable of enforcing a 10 Å layer-to-layer distance, todorokite will be formed; otherwise, the product of reaction will be cryptomelane.

Growth mechanisms proposed from previous experimental studies performed at higher temperatures were confirmed for conditions relevant to surface soil. The four-stage transformation begins with the migration of Mn^3+^ from layer to the interlayer to form ^TC^Mn^3+^ that further connect to hydration spheres belonging to ^TC^Mn^3+^ from adjacent layers. Crystals then grow first along the “tunnel” direction to form needle-like crystals. These crystals coalesce along their long dimension to form lath-like crystals which, in a final step, stack along c* with rotation by *n* × 120° between adjacent laths. Cryptomelane structure was observed only in these latter crystals (type 4), but is certainly present also in crystals type 2 and 3.

## Methods

### Samples investigated

Samples used for the present study are those studied by Grangeon and coworkers [49] and Manceau and coworkers [70]. They were synthesized using the redox method [2] and were left for equilibration at pH values ranging from 3 to 10 immediately after synthesis. They were then left to age for 10 years in the dark and in the dry state. For consistency with Grangeon and coworkers [49], samples are labeled MndBiXX_10y, where XX is the equilibrium pH and “10y” stands for “10 years of ageing”.

### X-ray diffraction pattern (XRD) analysis and modeling

XRD patterns were modeled using the software developed by Plançon [82], based on the formalism developed by Drits and Tchoubar [83]. This specific routine allows for the simulation of (lamellar) structures affected by various nature and density of layer defects (e.g., layer vacancies, isomorphic substitutions) and stacking defects (e.g., well-defined or random stacking faults, interstratification). It has been previously applied to the study of nanocrystalline manganese or iron oxides [69, 84, 85], nanocrystalline calcium silicate hydrates [86–88] and phyllosilicates [89, 90]. As discussed by Manceau and coworkers [70] the simulation of the [11, 20], [31, 02] and [22, 40] bands (using a C-centered hexagonal unit-cell, with *γ* = 90°) is sufficient to accurately determine the structure of synthetic phyllomanganates, and only these bands were modeled here. All structure parameters but crystallite size, *a* and *b* lattice parameters, abundance of ^TC^Mn^3+^, layer vacancies and of interlayer water molecules were kept identical to those determined by Grangeon and coworkers [49]. An example of the sensitivity of calculated XRD patterns to the number of ^TC^Mn^3+^ is available in the Additional File 2: Data S2. The cryptomelane pattern was calculated using the structure model from Vicat and coworkers [91], assuming a CSD size of 6 nm.

### High energy X-ray scattering coupled with pair distribution function (PDF) analysis and modeling

X-ray diffraction patterns were collected at ID15B high-energy beamline of the European Synchrotron Radiation Facility (ESRF, Grenoble, France), using energy of 87 keV and a PerkinElmer flat panel detector. Data were acquired on randomly-oriented powders packed in polyimide capillaries having a diameter of 1 mm and on empty capillary used for background subtraction. 40 frames of 5 s, corrected for detector’s dark current, were collected for each sample. After instrumental calibration using a NIST certified CeO_2_ powder sample, frames were integrated to one dimensional patterns [92] and averaged. Data were then transformed to PDF patterns using PdfGetX3 [93], and fit using PDFGui [94]. The model from Manceau and coworkers [70] was used to refine the patterns. The only modification was that ^TC^Mn^3+^ was allowed to sorb on both sides of a layer vacancy. The refined parameters were the abundance of ^TC^Mn^3+^, layer vacancies, lattice parameters and Debye–Waller factors. *q* broadening and *q* dampening factors were retrieved from simulation of a CeO_2_ pattern and found to be equal to, respectively, 0.044 and 0.048.

### Transmission electron microscopy (TEM)

TEM was performed using a Philips CM20 operated at 200 kV. Samples were deposited on a copper grid prior to observation. When samples were first dispersed in ethanol or in water, and deposited on the grid from the suspension, they were rapidly altered under the beam, with amorphous products occurring within a few seconds. To circumvent this problem, samples were first embedded in epoxy resin and left in the dark for 48 h until full polymerization. Cutting was performed using an ultramicrotome Reichert-Jung Ultra-cut E. Five to ten thin sections having thicknesses of about 100 nm were collected on the surface of the water contained in the boated knife and picked up on a lacey carbon film loaded on copper grids.

### Scanning transmission electron microscopy (STEM)

STEM experiments were performed using a Nion Ultra-STEM 200 operated at 100 kV. Sample preparation was identical to that applied for TEM measurements, except that the thickness of the slices containing the sample embedded in epoxy resin was reduced to ~50 nm. Image presented in the present study was acquired in high-angular annular dark-field (HAADF) mode. In order to ease visualization of the structural features, the raw image was Fourier-transformed and filtered using a band pass with a window set for spatial frequencies between 1.6 and 40 nm^−1^, and a color threshold was then applied to reduce the contribution from pixels having a grey color lower than half of the mean image grey value.

## Additional files

Additional file 1:
**Data S1.** A table listing all parameters, but those listed in Table 1, which were refined during PDF data analysis. Additional file 2:
**Data S2.** The sensitivity of calculated XRD patterns to the number of ^TC^Mn^3+^.
