# Supplementary material for: Cryptomelane formation from nanocrystalline vernadite precursor: a high energy X-ray scattering and transmission electron microscopy perspective on reaction mechanisms
Source: Geochem Trans. 2015 Sep 2;16:12. doi: 10.1186/s12932-015-0028-y (PMC4556320; doi:10.1186/s12932-015-0028-y)
Supplement: Additional file 1: — Data S1. A table listing all parameters, but those listed in Table 1, which were refined during PDF data analysis. [file 12932_2015_28_MOESM1_ESM.doc]

**Electronic Annex**

to the article:

“*Cryptomelane formation from nanocrystalline vernadite precursor: a high energy X-ray scattering and transmission electron microscopy perspective on reaction mechanisms*”

authored by Sylvain Grangeon, Alejandro Fernandez-Martinez, Fabienne Warmont, Alexandre Gloter, Nicolas Marty, Agnieszka Poulain & Bruno Lanson

Supplementary data 1: Anisotropic Debye-Waller factors (U), scale factor, correlated motion related quadratic term coefficient (Delta2) and particule size (Spdiameter) retrieved from analysis of PDF data.

| Species (if applicable) | Parameter |  |  | Sample |  |
| --- | --- | --- | --- | --- | --- |
|  |  | MndBi3_10y | MndBi4_10y | MndBi8_10y | MndBi10_10y |
| Mn | U(11) and U(22) (Å²) | 0.003 | 0.001 | 0.002 | 0.001 |
|  | U(33) (Å²) | 0.006 | 0.01 | 0.013 | 0.022 |
| Layer O and O from ^TC^Mn coordination sphere | U(11) and U(22) (Å²) | 0.001 | 0.003 | 0.003 | 0.004 |
|  | U(33) (Å²) | 0.074 | 0.045 | 0.07 | 0.061 |
| Na and interlayer H_2_O | U(11), U(22) and U(33) (Å²) | 0.001 | 0.004 | 0.001 | 0.001 |
|  |  |  |  |  |  |
|  | Scale factor | 1.43 | 1.45 | 1.43 | 1.36 |
|  | Delta2 | 2.85 | 2.53 | 3.17 | 2.93 |
|  | Spdiameter (Å) | 18.91 | 17.13 | 17.31 | 18.33 |
| Note: Delta2 and U(33) from O suffer from uncertainties, as they were correlated. | | | | | |
