# Supplementary material for: Cryptomelane formation from nanocrystalline vernadite precursor: a high energy X-ray scattering and transmission electron microscopy perspective on reaction mechanisms
Source: Geochem Trans. 2015 Sep 2;16:12. doi: 10.1186/s12932-015-0028-y (PMC4556320; doi:10.1186/s12932-015-0028-y)

**Electronic Annex**

to the article:

“*Cryptomelane formation from nanocrystalline vernadite precursor: a high energy X-ray scattering and transmission electron microscopy perspective on reaction mechanisms*”

authored by Sylvain Grangeon, Alejandro Fernandez-Martinez, Fabienne Warmont, Alexandre Gloter, Nicolas Marty, Agnieszka Poulain & Bruno Lanson

Supplementary data 2: Example of the sensitivity of calculated XRD patterns to the number of interlayer ^TC^Mn^3+^. The experimental pattern from MndBi8_10y is the black solid line and the red solid line is the best simulation (in which the abundance of ^TC^Mn^3+^ is 0.13 per layer octahedron). Solid green line: ^TC^Mn^3+^ reduced to 0.08 per layer octahedron. Solid brown line: ^TC^Mn^3+^ increased to 0.18 per layer octahedron. All other parameters remained identical in all simulations.

Only the [11, 20] band was calculated, as it is the most sensitive to the number of interlayer species sorbed in a TC configuration.


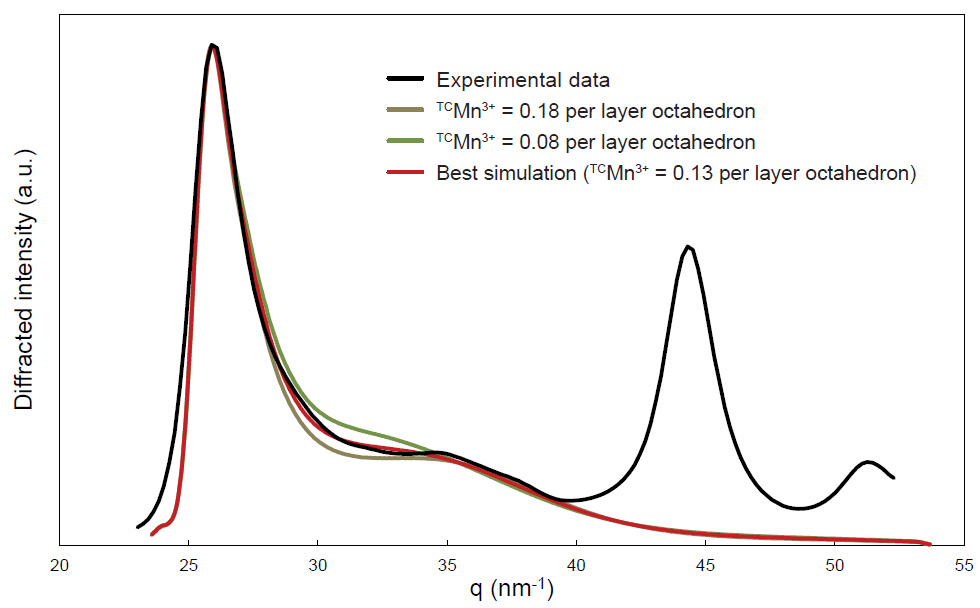

Supplement: Additional file 2: — Data S2. The sensitivity of calculated XRD patterns to the number of TCMn3+. [file 12932_2015_28_MOESM2_ESM.doc]
